# Supplementary figures and images for: Comparative study of gut microbiota in Tibetan wild asses (Equus kiang) and domestic donkeys (Equus asinus) on the Qinghai-Tibet plateau
Source: PeerJ. 2020 Jun 4;8:e9032. doi: 10.7717/peerj.9032 (PMC7276150; doi:10.7717/peerj.9032)

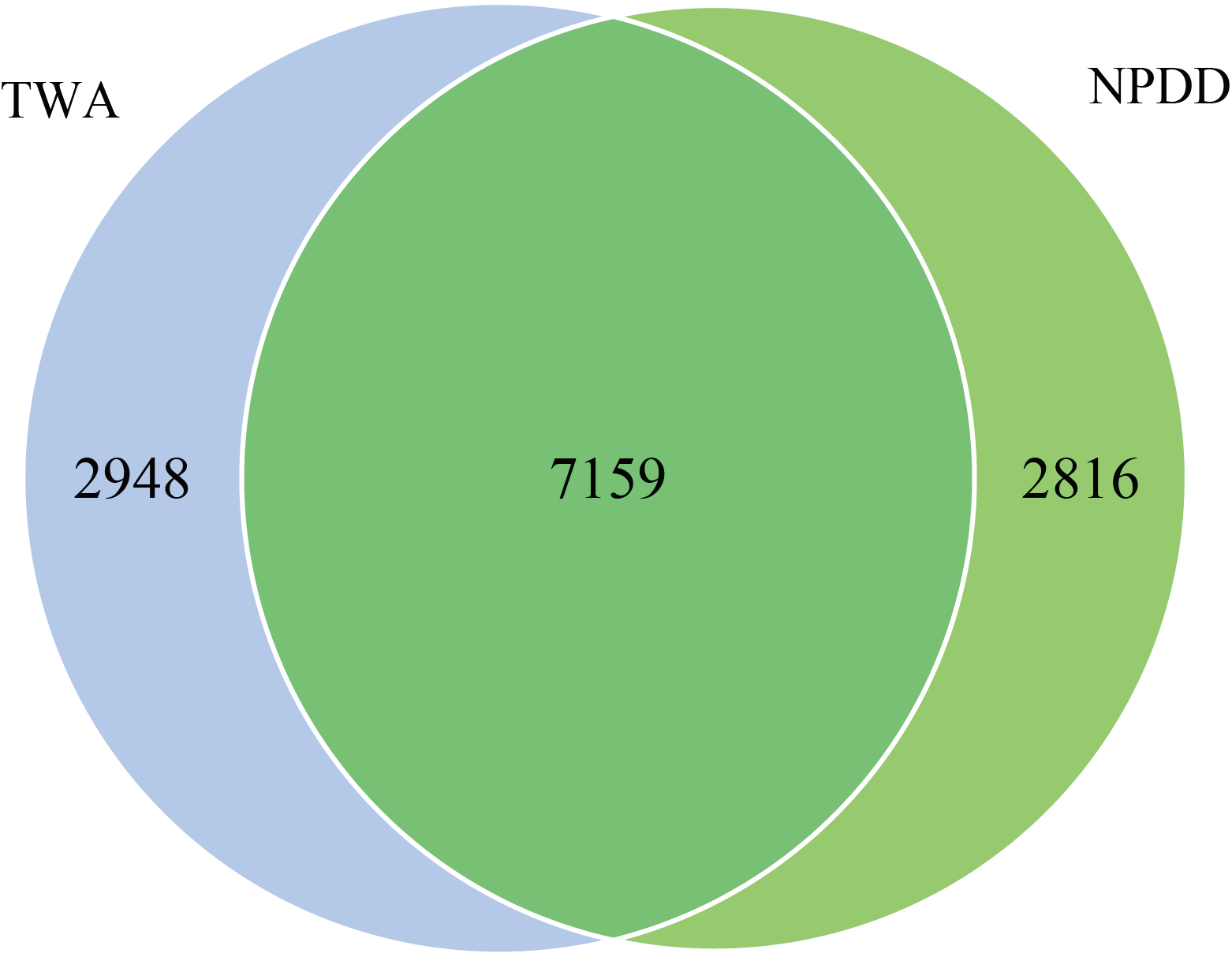

Supplement: Figure S1 — TWA=Tibetan wild asses, NPDD=natural pasture domestic donkeys. [file peerj-08-9032-s001.png]

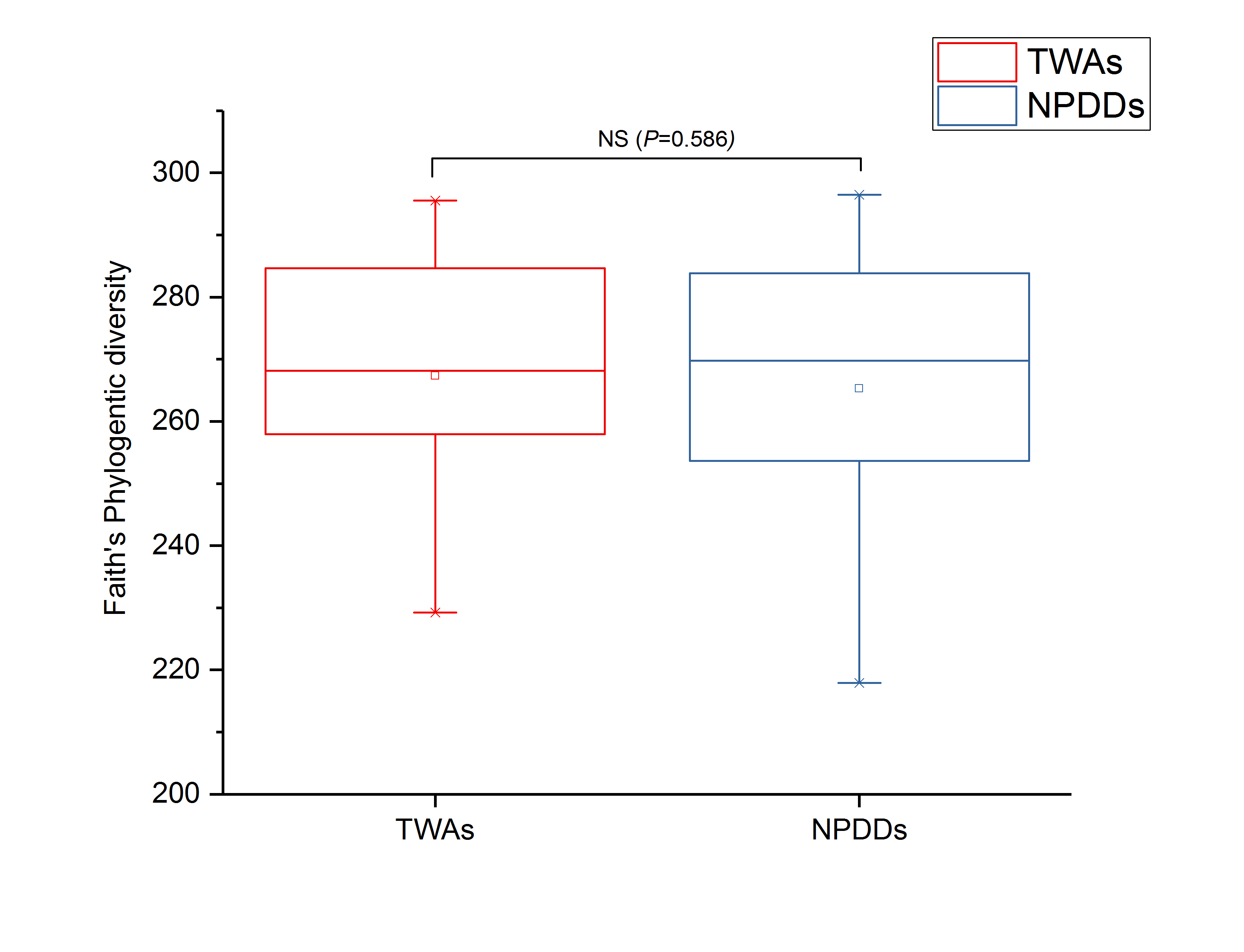

Supplement: Figure S2 — The box represents the interquartile range between the first and third quartiles, and the symbol ”—” represents the max value, ” ×” represents the variation range from 1% to 99% and ” □” represents the mean value.TWAs= Tibetan wild asses, NPDDs= natural pasture domestic donkeys. [file peerj-08-9032-s002.png]

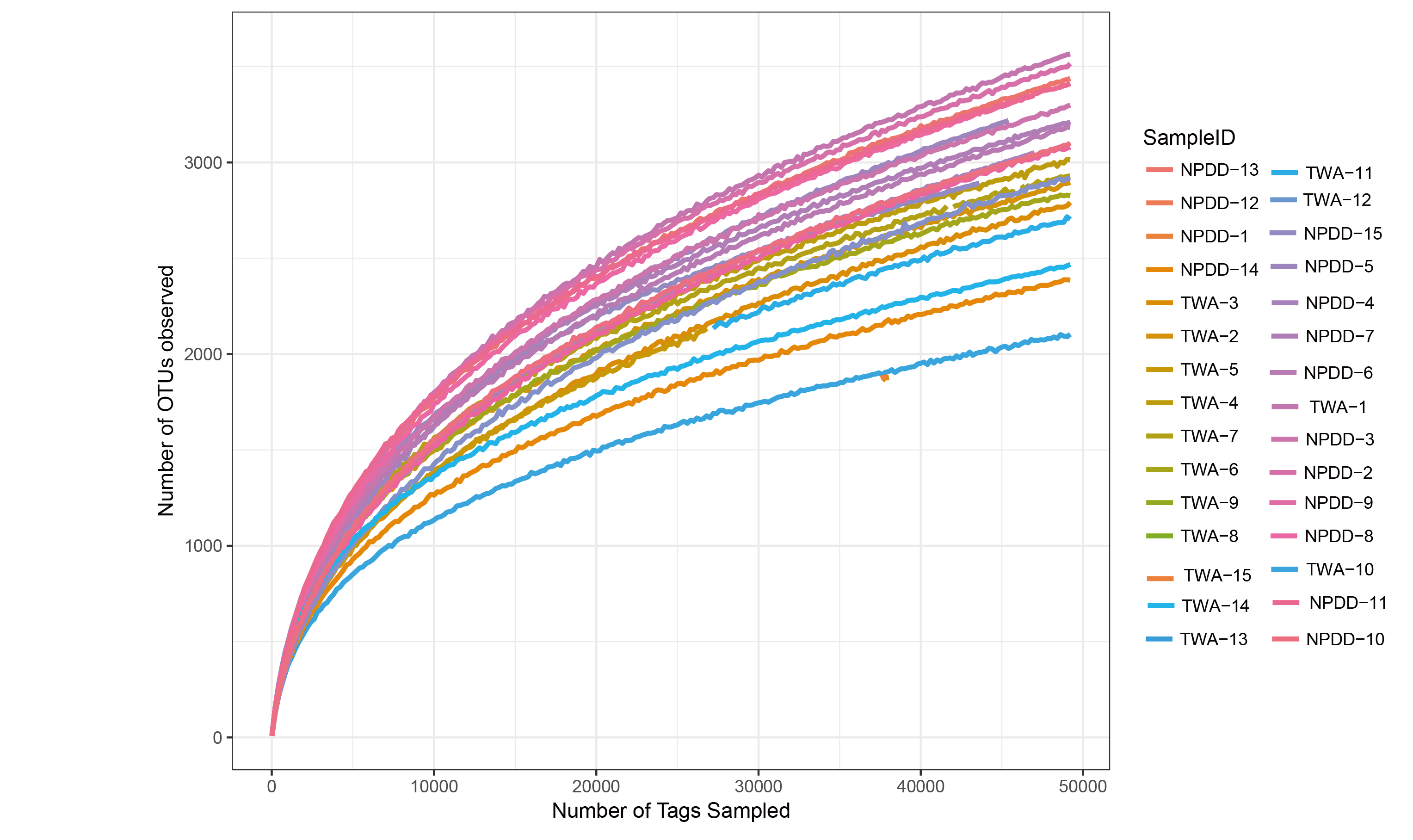

Supplement: Figure S3 — Each line represents a single animal. OTUs in this analysis were defined at 97% similarity. TWA=Tibetan wild assed, NPDD=natural pasture domestic donkeys. [file peerj-08-9032-s003.png]

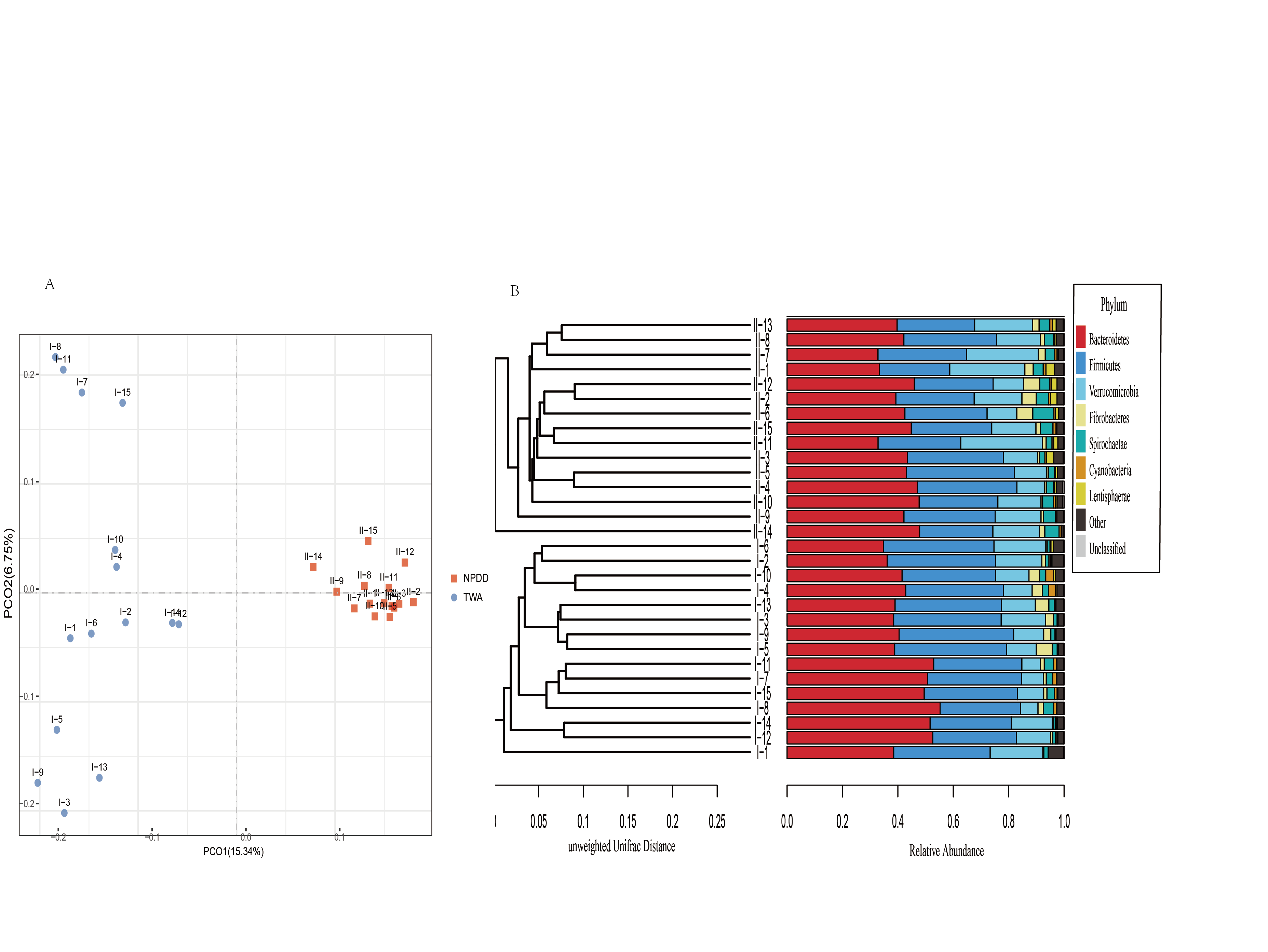

Supplement: Figure S4 — (A) PCoA scatter plot. (B) Unweight pair-group method with arithmetic means (UPGMA) with hierarchical tree and bacterial relative abundance at the phylum level. TWAs=Tibetan wild asses, NPDDs=natural pasture domestic donkeys. [file peerj-08-9032-s004.png]

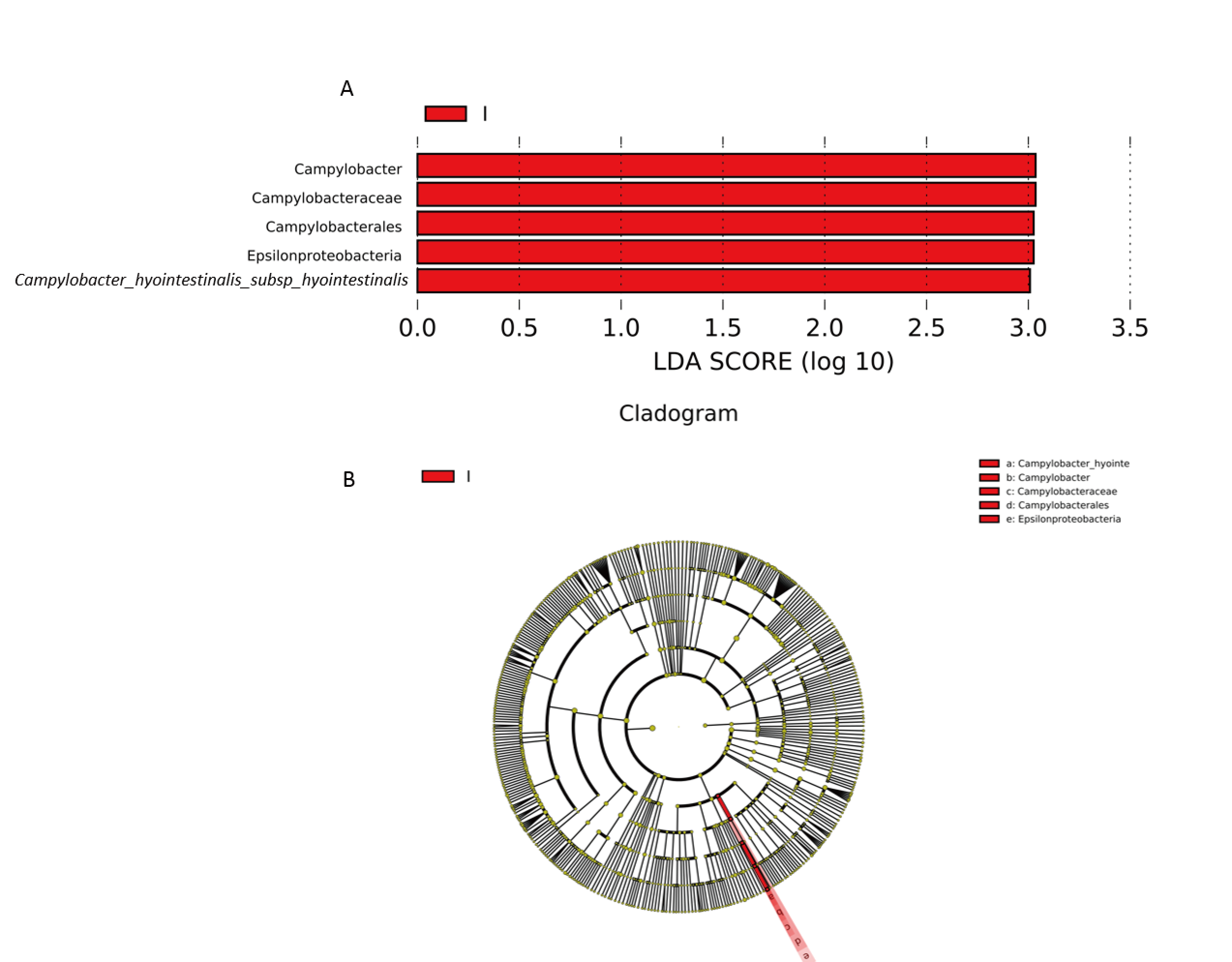

Supplement: Figure S5 — (A) The LEfSe bar representing significant biomarkers between TWA and NPDD. (B) The cladogram between TWA and NPDD. P ¡ 0.05, LDA value ¡ 2. Each genus that does not share annotations was significantly different (P ¡ 0.05). TWA=Tibetan wild assed, NPDD=natural pasture domestic donkeys. [file peerj-08-9032-s005.png]
