# Supplementary material for: Comparative study of gut microbiota in Tibetan wild asses (Equus kiang) and domestic donkeys (Equus asinus) on the Qinghai-Tibet plateau
Source: PeerJ. 2020 Jun 4;8:e9032. doi: 10.7717/peerj.9032 (PMC7276150; doi:10.7717/peerj.9032)
Supplement: Table S1 — TWAs= Tibetan wild asses, NPDDs= natural pasture domestic donkeys. [file peerj-08-9032-s006.docx]

| Groups | Sample Name | Raw PE | Clean PE | Raw Tags | Clean Tags | Effective Tags | Effective Ratio (%) |
| --- | --- | --- | --- | --- | --- | --- | --- |
| TWA | I-1 | 115435 | 111589 | 111017 | 110436 | 109909 | 95.21 |
|  | I-2 | 110821 | 107318 | 106682 | 106175 | 104678 | 94.46 |
|  | I-3 | 124091 | 119905 | 119279 | 118650 | 116421 | 93.82 |
|  | I-4 | 98081 | 94836 | 94259 | 93839 | 92863 | 94.68 |
|  | I-5 | 97168 | 93985 | 93434 | 92931 | 91962 | 94.64 |
|  | I-6 | 89116 | 86181 | 85718 | 85292 | 84886 | 95.25 |
|  | I-7 | 127789 | 123629 | 122978 | 122258 | 121142 | 94.8 |
|  | I-8 | 91152 | 87965 | 87432 | 86969 | 86541 | 94.94 |
|  | I-9 | 108212 | 104214 | 103614 | 103073 | 101827 | 94.1 |
|  | I-10 | 96495 | 93257 | 92724 | 92279 | 91372 | 94.69 |
|  | I-11 | 102423 | 98772 | 98181 | 97686 | 97212 | 94.91 |
|  | I-12 | 99659 | 96305 | 95798 | 95239 | 93542 | 93.86 |
|  | I-13 | 68077 | 65781 | 65409 | 65018 | 63510 | 93.29 |
|  | I-14 | 101382 | 97819 | 97349 | 96842 | 95022 | 93.73 |
|  | I-15 | 73192 | 70454 | 70011 | 69632 | 69029 | 94.31 |
| NPDD | II-1 | 78881 | 76208 | 75839 | 75505 | 74394 | 94.31 |
|  | II-2 | 72258 | 69794 | 69410 | 69064 | 67888 | 93.95 |
|  | II-3 | 81749 | 79053 | 78613 | 78215 | 76788 | 93.93 |
|  | II-4 | 62255 | 59969 | 59684 | 59345 | 58165 | 93.43 |
|  | II-5 | 68751 | 66564 | 66245 | 65907 | 63744 | 92.72 |
|  | II-6 | 74066 | 71693 | 71296 | 70949 | 69700 | 94.11 |
|  | II-7 | 68372 | 66029 | 65713 | 65412 | 64381 | 94.16 |
|  | II-8 | 63156 | 60989 | 60591 | 60333 | 59073 | 93.54 |
|  | II-9 | 67787 | 65648 | 65286 | 64954 | 64593 | 95.29 |
|  | II-10 | 71197 | 68929 | 68664 | 68314 | 66824 | 93.86 |
|  | II-11 | 65908 | 63901 | 63592 | 63280 | 62416 | 94.7 |
|  | II-12 | 67965 | 65779 | 65473 | 65111 | 63921 | 94.05 |
|  | II-13 | 73607 | 70789 | 70317 | 69980 | 68698 | 93.33 |
|  | II-14 | 69379 | 67067 | 66679 | 66354 | 65862 | 94.93 |
|  | II-15 | 52248 | 50434 | 50172 | 49950 | 49504 | 94.75 |
|  | Total | 2540672 | 2454856 | 2441459 | 2428992 | 2395867 |  |
