# Supplementary material for: Comparative study of gut microbiota in Tibetan wild asses (Equus kiang) and domestic donkeys (Equus asinus) on the Qinghai-Tibet plateau
Source: PeerJ. 2020 Jun 4;8:e9032. doi: 10.7717/peerj.9032 (PMC7276150; doi:10.7717/peerj.9032)
Supplement: Table S2 — TWAs= Tibetan wild asses, NPDDs= natural pasture domestic donkeys. [file peerj-08-9032-s007.docx]

| Phylum | TWAs | NPDDs | *P* value |
| --- | --- | --- | --- |
| *Bacteroidetes* | 44.21±1.82 | 41.66±1.33 | 0.035 |
| *Firmicutes* | 35.32±1.07 | 30.75±0.97 | 0.043 |
| *Verrucomicrobia* | 12.28±1.05 | 17.16±1.59 | 0.218 |
| *Fibrobacteres* | 2.16±0.44 | 2.28±0.48 | 0.870 |
| *Spirochaetae* | 1.91±0.22 | 3.78±0.37 | 0.305 |
| *Proteobacteria* | 0.95±0.09 | 0.97±0.10 | 0.635 |
| *Cyanobacteria* | 0.84±0.21 | 0.65±0.06 | 0.004 |
| *Lentisphaerae* | 0.34±0.06 | 1.18±0.25 | 0.000 |
| *Synergistetes* | 0.64±0.10 | 0.24±0.04 | 0.009 |
| *Planctomycetes* | 0.24±0.05 | 0.29±0.06 | 0.424 |
