# Supplementary material for: Comparative study of gut microbiota in Tibetan wild asses (Equus kiang) and domestic donkeys (Equus asinus) on the Qinghai-Tibet plateau
Source: PeerJ. 2020 Jun 4;8:e9032. doi: 10.7717/peerj.9032 (PMC7276150; doi:10.7717/peerj.9032)
Supplement: Table S3 — TWAs= Tibetan wild asses, NPDDs= natural pasture domestic donkeys. [file peerj-08-9032-s008.docx]

| Genus | TWAs | NPDDs | *P* value |
| --- | --- | --- | --- |
| *Rikenellaceae_RC9_gut_group* | 8.09±0.26 | 8.62±0.41 | 0.103 |
| *Fibrobacter* | 2.16±0.44 | 2.07±0.44 | 0.628 |
| *Treponema_2* | 1.81±0.21 | 3.70±0.37 | 0.247 |
| *Lachnospiraceae_AC2044_group* | 1.20**±**0.10 | 2.74±0.63 | 0.011 |
| *Ruminococcaceae_NK4A214_group* | 3.01±0.34 | 1.96±0.11 | 0.002 |
| *Ruminococcaceae_UCG_010* | 2.76±0.13 | 2.24±0.09 | 0.258 |
| *Phascolarctobacterium* | 1.98±0.21 | 1.20±0.10 | 0.012 |
| *Christensenellaceae_R_7_group* | 1.65±0.26 | 1.44±0.14 | 0.064 |
| *Coprostanoligenes_group* | 2.16±0.27 | 1.09±0.07 | 0.000 |
| *Prevotellaceae_UCG_001* | 1.91±0.26 | 1.24±0.18 | 0.070 |
| *Lachnospiraceae_XPB1014_group* | 1.26±0.22 | 0.80±0.06 | 0.010 |
| *Anaerovorax* | 1.41±0.10 | 1.08±0.10 | 0.055 |
| *Prevotellaceae_UCG_003* | 1.14±0.10 | 1.31±0.11 | 0.326 |
| *Akkermansia* | 1.75±0.59 | 0.43±0.11 | 0.000 |
| *Prevotellaceae_UCG_004* | 0.91±0.10 | 1.08±0.12 | 0.668 |
