# Supplementary material for: Comparative study of gut microbiota in Tibetan wild asses (Equus kiang) and domestic donkeys (Equus asinus) on the Qinghai-Tibet plateau
Source: PeerJ. 2020 Jun 4;8:e9032. doi: 10.7717/peerj.9032 (PMC7276150; doi:10.7717/peerj.9032)
Supplement: Table S4 — TWAs= Tibetan wild asses, NPDDs= natural pasture domestic donkeys. [file peerj-08-9032-s009.docx]

| KEEG pathways (Level 2) | Groups (%) | | P values |
| --- | --- | --- | --- |
|  | TWAs | NPDDs |  |
| Folding, Sorting and Degradation | 2.724±0.053 | 2.752±0.062 | 0.209 |
| Amino Acid Metabolism | 11.667±0.200 | 11.247±0.192 | 0.000 |
| Biosynthesis of Other Secondary Metabolites | 0.889±0.013 | 0.873±0.020 | 0.035 |
| Cancers | 0.109±0.002 | 0.113±0.004 | 0.001 |
| Carbohydrate Metabolism | 14.434±0.143 | 14.519±0.140 | 0.125 |
| Cardiovascular Diseases | 0.001±1.5 | 0.002±0.000 | 0.000 |
| Cell Communication | 0.001±0.000 | 0.001±0.000 | 0.005 |
| Cell Growth and Death | 1.784±0.039 | 1.808±0.029 | 0.080 |
| Cell Motility | 2.296±0.146 | 2.539±0.129 | 0.000 |
| Circulatory System | 0.004±0.001 | 0.004±0.000 | 0.001 |
| Digestive System | 0.235±0.027 | 0.233±0.019 | 0.823 |
| Endocrine and Metabolic Diseases | 0.064±0.003 | 0.064±0.002 | 0.657 |
| Endocrine System | 0.363±0.0121 | 0.381±0.009 | 0.000 |
| Energy Metabolism | 6.990±0.113 | 6.832±0.076 | 0.000 |
| Environmental Adaptation | 0.261±0.009 | 0.265±0.006 | 0.225 |
| Excretory System | 0.029±0.003 | 0.026±0.001 | 0.004 |
| Glycan Biosynthesis and Metabolism | 3.535±0.269 | 3.335±0.090 | 0.017 |
| Immune Diseases | 0.037±0.004 | 0.042±0.002 | 0.001 |
| Immune System | 0.079±0.006 | 0.082±0.005 | 0.272 |
| Infectious Diseases | 1.557±0.041 | 1.580±0.022 | 0.085 |
| Lipid Metabolism | 2.913±0.070 | 2.871±0.045 | 0.072 |
| Membrane Transport | 10.060±0.213 | 10.572±0.302 | 0.000 |
| Metabolism of Cofactors and Vitamins | 6.752±0.114 | 6.753±0.066 | 0.970 |
| Metabolism of Other Amino Acids | 2.152±0.057 | 2.171±0.021 | 0.275 |
| Metabolism of Terpenoids and Polyketides | 2.003±0.026 | 2.016±0.028 | 0.243 |
| Nervous System | 0.142±0.003 | 0.137±0.006 | 0.018 |
| Neurodegenerative Diseases | 0.112±0.005 | 0.108±0.003 | 0.011 |
| Nucleotide Metabolism | 6.570±0.084 | 6.564±0.078 | 0.842 |
| Replication and Repair | 5.728±0.087 | 5.724±0.124 | 0.923 |
| Sensory System | 2.192E-07±1.2E-05 | 2.652±0.000 | 0.337 |
| Signal Transduction | 7.274±0.232 | 7.305±0.217 | 0.716 |
| Signaling Molecules and Interaction | 0.001±0.000 | 0.001±0.000 | 0.014 |
| Substance Dependence | 0.010±0.002 | 0.014±0.002 | 0.000 |
| Transcription | 0.286±0.011 | 0.290±0.013 | 0.354 |
| Translation | 6.015±0.108 | 5.956±0.104 | 0.152 |
| Transport and Catabolism | 0.385±0.068 | 0.360±0.029 | 0.230 |
| Xenobiotics Biodegradation and Metabolism | 2.536±0.026 | 2.464±0.049 | 0.000 |
