# Supplementary material for: Comparative study of gut microbiota in Tibetan wild asses (Equus kiang) and domestic donkeys (Equus asinus) on the Qinghai-Tibet plateau
Source: PeerJ. 2020 Jun 4;8:e9032. doi: 10.7717/peerj.9032 (PMC7276150; doi:10.7717/peerj.9032)
Supplement: Table S5 — TWAs= Tibetan wild asses, NPDDs= natural pasture domestic donkeys, VIP= Variable importance in the projection. [file peerj-08-9032-s010.docx]

| ID | Groups (%) | | VIP | P value |
| --- | --- | --- | --- | --- |
|  | TWAs | NPDDs |  |  |
| Citrate cycle (TCA cycle) | 0.496 | 0.457 | 1.999 | 0.000 |
| Pentose and glucuronate interconversions | 0.854 | 0.884 | 1.431 | 0.028 |
| Fructose and mannose metabolism | 1.760 | 1.795 | 1.720 | 0.016 |
| Photosynthesis | 0.260 | 0.281 | 1.146 | 0.025 |
| Glycine, serine and threonine metabolism | 1.286 | 1.223 | 2.208 | 0.000 |
| Cysteine and methionine metabolism | 1.444 | 1.399 | 2.268 | 0.000 |
| Valine, leucine and isoleucine degradation | 0.395 | 0.350 | 1.938 | 0.000 |
| Geraniol degradation | 0.121 | 0.112 | 1.056 | 0.001 |
| Valine, leucine and isoleucine biosynthesis | 0.573 | 0.549 | 1.296 | 0.001 |
| Lysine biosynthesis | 1.138 | 1.086 | 2.453 | 0.000 |
| Arginine and proline metabolism | 1.856 | 1.803 | 2.587 | 0.031 |
| Histidine metabolism | 1.035 | 0.992 | 2.302 | 0.000 |
| Tyrosine metabolism | 0.495 | 0.481 | 1.113 | 0.004 |
| Phenylalanine metabolism | 0.446 | 0.418 | 1.818 | 0.002 |
| Benzoate degradation | 0.465 | 0.441 | 1.585 | 0.000 |
| Phenylalanine, tyrosine and tryptophan biosynthesis | 1.260 | 1.221 | 1.705 | 0.001 |
| Starch and sucrose metabolism | 2.856 | 2.973 | 2.683 | 0.008 |
| Amino sugar and nucleotide sugar metabolism | 2.525 | 2.571 | 1.547 | 0.020 |
| Lipopolysaccharide biosynthesis | 0.577 | 0.479 | 2.977 | 0.000 |
| Inositol phosphate metabolism | 0.186 | 0.199 | 1.181 | 0.000 |
| Pyruvate metabolism | 1.155 | 1.111 | 1.861 | 0.000 |
| Glyoxylate and dicarboxylate metabolism | 0.767 | 0.735 | 1.726 | 0.000 |
| Nitrotoluene degradation | 0.145 | 0.128 | 1.235 | 0.042 |
| Propanoate metabolism | 0.394 | 0.374 | 1.365 | 0.000 |
| Butanoate metabolism | 0.665 | 0.639 | 1.812 | 0.000 |
| One carbon pool by folate | 0.571 | 0.553 | 1.377 | 0.000 |
| Carbon fixation pathways in prokaryotes | 1.052 | 0.985 | 2.691 | 0.000 |
| Riboflavin metabolism | 0.366 | 0.351 | 1.284 | 0.000 |
| Pantothenate and CoA biosynthesis | 0.800 | 0.779 | 1.355 | 0.000 |
| Folate biosynthesis | 0.514 | 0.496 | 1.146 | 0.001 |
| Atrazine degradation | 0.100 | 0.087 | 1.072 | 0.000 |
| Porphyrin and chlorophyll metabolism | 1.543 | 1.648 | 3.065 | 0.000 |
| Nitrogen metabolism | 1.601 | 1.560 | 1.947 | 0.002 |
| ABC transporters | 8.544 | 9.068 | 6.682 | 0.000 |
| Bacterial chemotaxis | 1.254 | 1.385 | 3.322 | 0.000 |
| Flagellar assembly | 1.042 | 1.153 | 2.972 | 0.000 |
| Phosphotransferase system (PTS) | 0.347 | 0.373 | 1.164 | 0.024 |
| Bacterial secretion system | 1.169 | 1.131 | 1.687 | 0.031 |
| Base excision repair | 0.617 | 0.597 | 1.487 | 0.000 |
| Meiosis - yeast | 0.117 | 0.136 | 1.101 | 0.005 |
