# Supplementary material for: Comparative study of gut microbiota in Tibetan wild asses (Equus kiang) and domestic donkeys (Equus asinus) on the Qinghai-Tibet plateau
Source: PeerJ. 2020 Jun 4;8:e9032. doi: 10.7717/peerj.9032 (PMC7276150; doi:10.7717/peerj.9032)
Supplement: Table S6 — TWAs= Tibetan wild asses, NPDDs= natural pasture domestic donkeys. [file peerj-08-9032-s011.docx]

| ID | metabolites pathways | Groups (%) | |
| --- | --- | --- | --- |
|  |  | TWAs | NPDDs |
| ko02010 | ABC transporters | 8.544±0.250 | 9.068±0.280 |
| ko02020 | Two-component system | 7.188±0.233 | 7.22±0.218 |
| ko00230 | Purine metabolism | 3.731±0.051 | 3.729±0.055 |
| ko00500 | Starch and sucrose metabolism | 2.856±0.106 | 2.973±0.108 |
| ko00970 | Aminoacyl-tRNA biosynthesis | 3.464±0.051 | 3.437±0.068 |
| ko00240 | Pyrimidine metabolism | 2.839±0.041 | 2.835±0.039 |
| ko00520 | Amino sugar and nucleotide sugar metabolism | 2.525±0.052 | 2.571±0.047 |
| ko03010 | Ribosome | 2.191±0.046 | 2.159±0.038 |
| ko03440 | Homologous recombination | 1.616±0.066 | 1.637±0.068 |
| ko00051 | Fructose and mannose metabolism | 1.76±0.031 | 1.795±0.041 |
| ko00550 | Peptidoglycan biosynthesis | 1.784±0.021 | 1.791±0.022 |
| ko00330 | Arginine and proline metabolism | 1.856±0.070 | 1.803±0.052 |
| ko04112 | Cell cycle - Caulobacter | 1.609±0.038 | 1.617±0.020 |
| ko00910 | Nitrogen metabolism | 1.601±0.022 | 1.56±0.039 |
| ko00190 | Oxidative phosphorylation | 1.5±0.038 | 1.489±0.030 |
| ko03018 | RNA degradation | 1.374±0.054 | 1.384±0.053 |
| ko00270 | Cysteine and methionine metabolism | 1.444±0.029 | 1.399±0.031 |
| ko00680 | Methane metabolism | 1.763±0.096 | 1.704±0.058 |
| ko03420 | Nucleotide excision repair | 1.386±0.029 | 1.373±0.040 |
| ko00860 | Porphyrin and chlorophyll metabolism | 1.543±0.060 | 1.648±0.060 |
